# Supplementary material for: An in situ hydrogel-mediated chemo-immunometabolic cancer therapy
Source: Nat Commun. 2022 Jul 2;13:3821. doi: 10.1038/s41467-022-31579-8 (PMC9250515; doi:10.1038/s41467-022-31579-8)

Supplementary Information for
An *in situ* hydrogel-mediated chemo-immunometabolic cancer therapy

Bo Wang[1*#], Jing Chen[1,2*], Julia S. Caserto[3*], Xi Wang[1], Minglin Ma[1#]

1. Department of Biological and Environmental Engineering, Cornell University, Ithaca, NY, USA
2. College of pharmacy, Nanjing University of Chinese Medicine, Nanjing 210023, China (present address)
3. Robert Frederick Smith School of Chemical and Biomolecular Engineering, Cornell University, Ithaca, NY, USA

Supplementary Fig. 1. Spearman correlation between TDO2 expression and T cell infiltration in human TNBC and melanoma, related to Fig. 1. Correlations of TDO2 expression with T cell markers CD3E and CD8A from the TCGA database are shown for TNBC (a, n = 1085) and SKCM (b, n = 558).

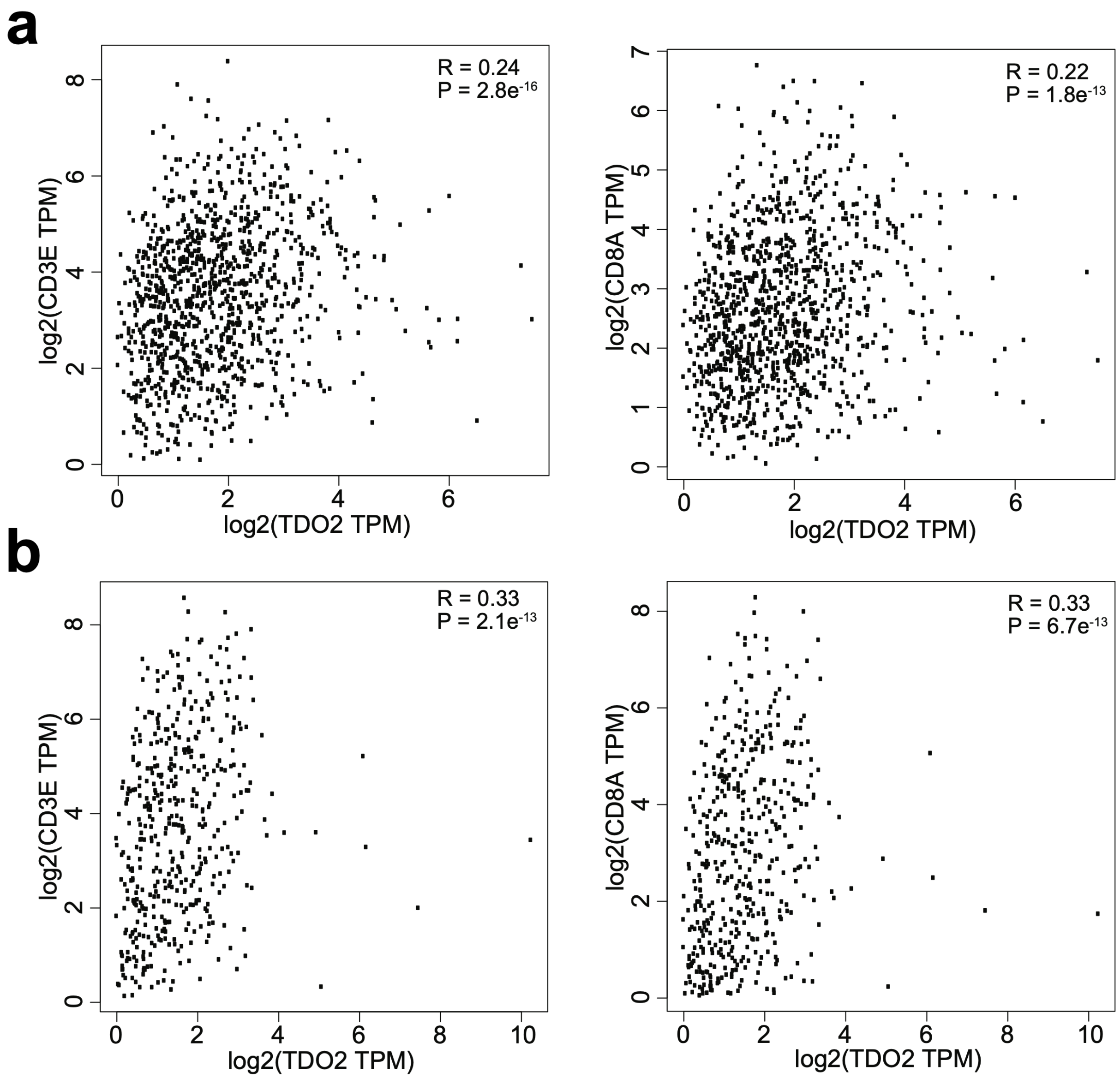

Supplementary Fig. 2. Spearman correlation between IDO1 and TDO2 expressions and T cell infiltration in other types of human cancers, ranked by their immunogenicity. Correlations of IDO1 (a) and TDO2 (b) expressions with T cell markers CD3E and CD8A from the TCGA database are shown. DLBC, lymphoid neoplasm diffuse large B-cell lymphoma (n = 47); COAD, colon adenocarcinoma (n = 275); HNSC, head and neck squamous cell carcinoma (n = 518); STAD, stomach adenocarcinoma (n = 407); OV, ovarian serous cystadenocarcinoma (n = 426); TGCT, testicular germ cell tumors (n = 136); LGG, brain lower grade glioma (n = 517).

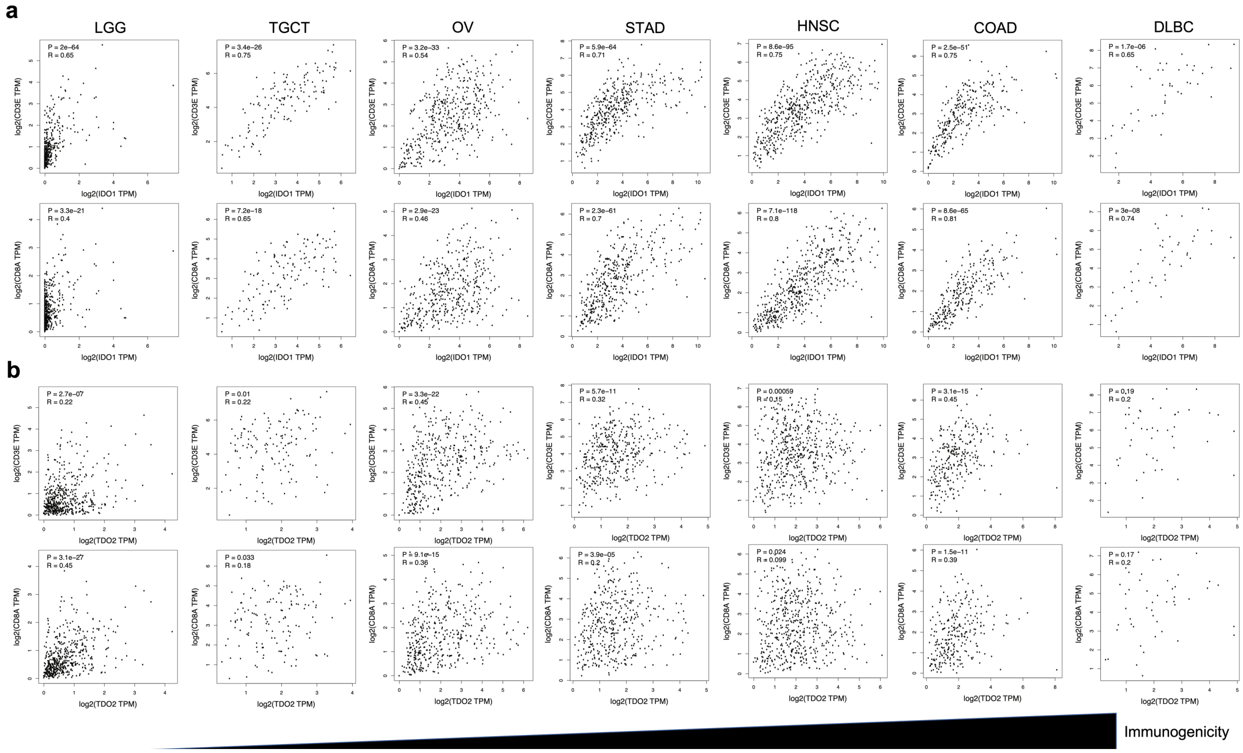

Supplementary Fig. 3. KYNase loading into the hydrogel. a, SDS-PAGE of purified KYNase. The experiment was performed once. **b,** Time needed to degrade 1mM Kyn by 1μM KYNase as free enzyme or encapsulated into 10% or 20% hydrogels, respectively. n = 3 biologically independent samples per group. Representative data from one of two experiments are shown, and data are presented as mean ± s.e.m.. Source data are provided as a Source Data file.

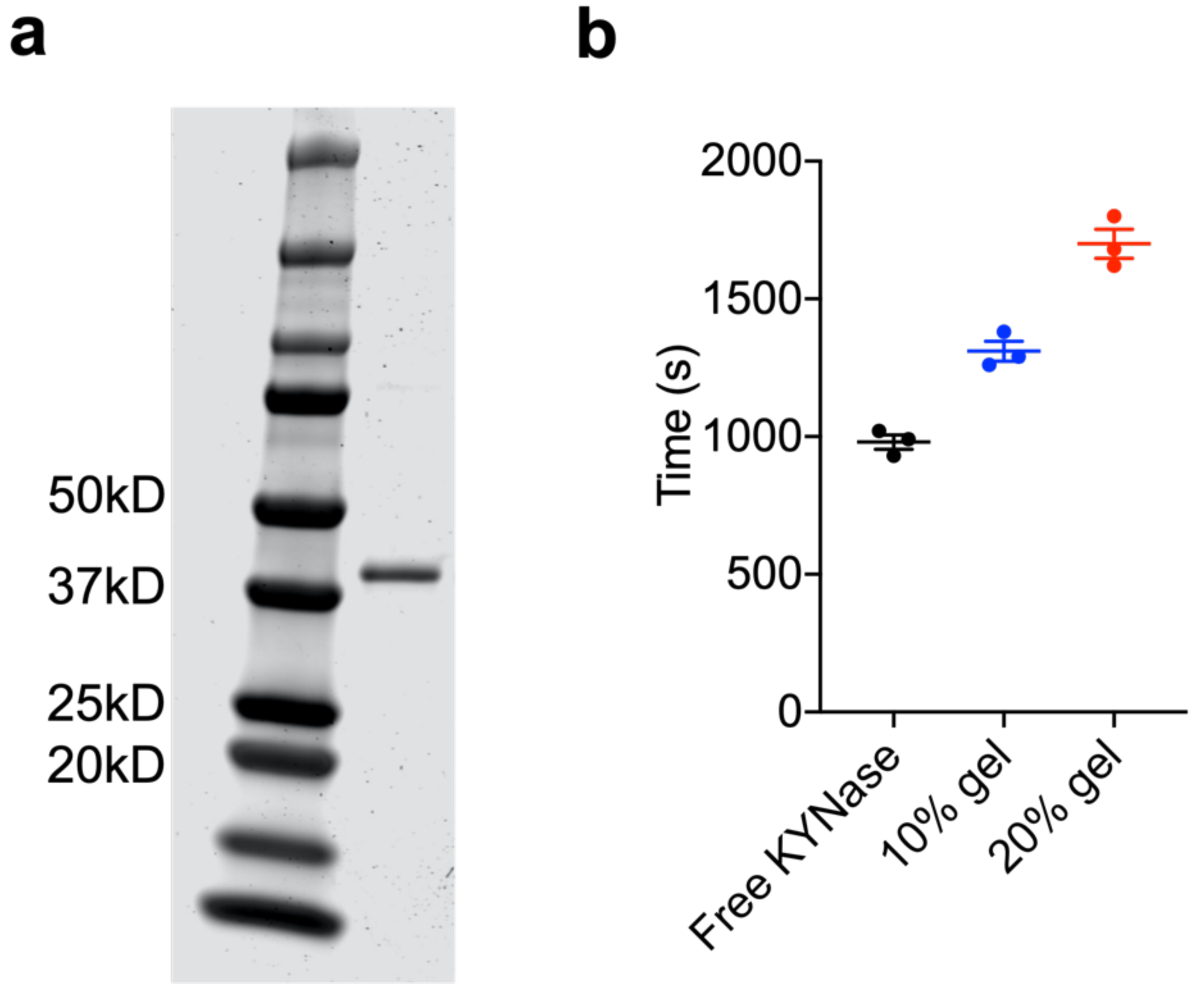

Supplementary Fig. 4. KYNase-loaded hydrogel delays 4T1 TNBC growth. 4T1 tumor cells were injected subcutaneously on day 0. Mice were treated with PBS, free KYNase, free KYNase administered as 3 doses, KYNase-loaded 10% and 20% hydrogels, respectively, on day 6 when tumor volumes reached 50mm^3. **a,** Average tumor growth over time. n = 5 mice for PBS, n = 6 mice for free KYNase groups, and n = 7 mice for KYNase loaded hydrogel groups. **b,** Survival curves for all treatment groups. Data are represented as mean ± s.e.m, and statistical significance was determined by two-way ANOVA with Turkey's post hoc test (a) or log-rank (Mantel-Cox) test (b). Source data are provided as a Source Data file.

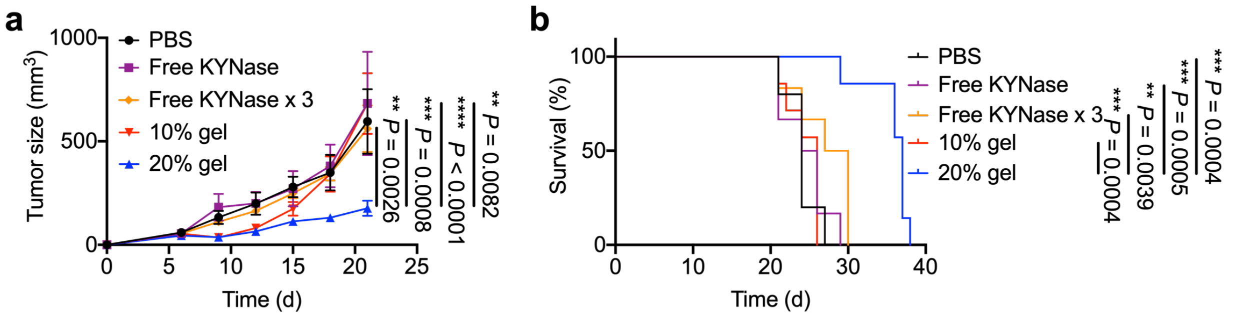

Supplementary Fig. 5. Histological analysis of tumor tissues on day 21 from 4T1 tumor-bearing mice that received different treatments (KYNase loaded 20% hydrogel (n = 7), KYNase loaded 10% hydrogel (n = 7), free KYNase (n = 6), PBS (n = 5) and free KYNase administered as 3 doses (n = 6), respectively), with enlargements of the regions indicated by dashed lines. Representative images from one mouse were shown. Scale bar, 100μm. The experiment was performed once.

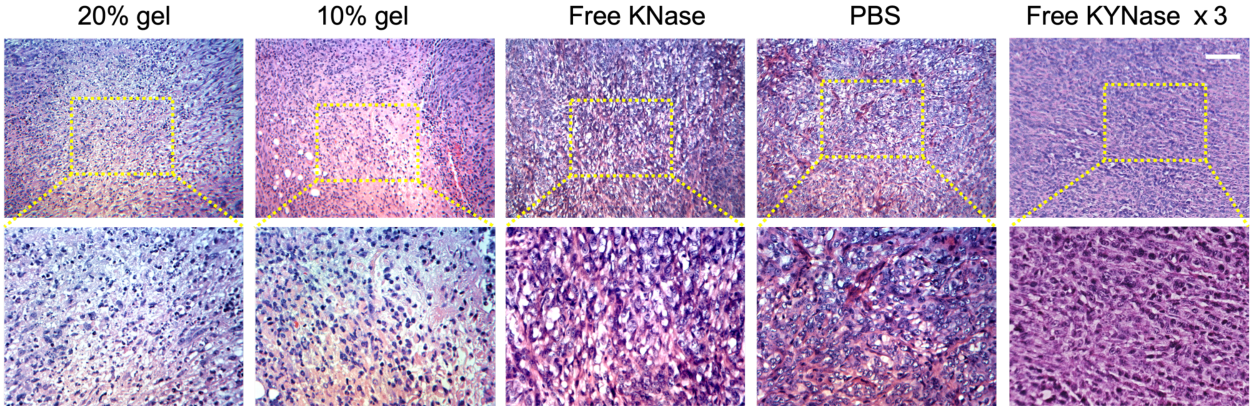

Supplementary Fig. 6. Safety of KYNase-loaded hydrogels for 4T1 TNBC treatment. **a,** Body weight changes of different treatment groups of mice. **b,** H&E staining of major organs from differently treated mice harvested on day 21. n = 5 mice for PBS, n =6 mice for free KYNase groups, and n = 7 mice for KYNase-loaded hydrogel groups. Red arrows indicate metastasis. Representative images from one mouse were shown. Scale bar, 100μm. Data are presented as mean ± s.e.m.. The experiment was performed once. Source data are provided as a Source Data file.

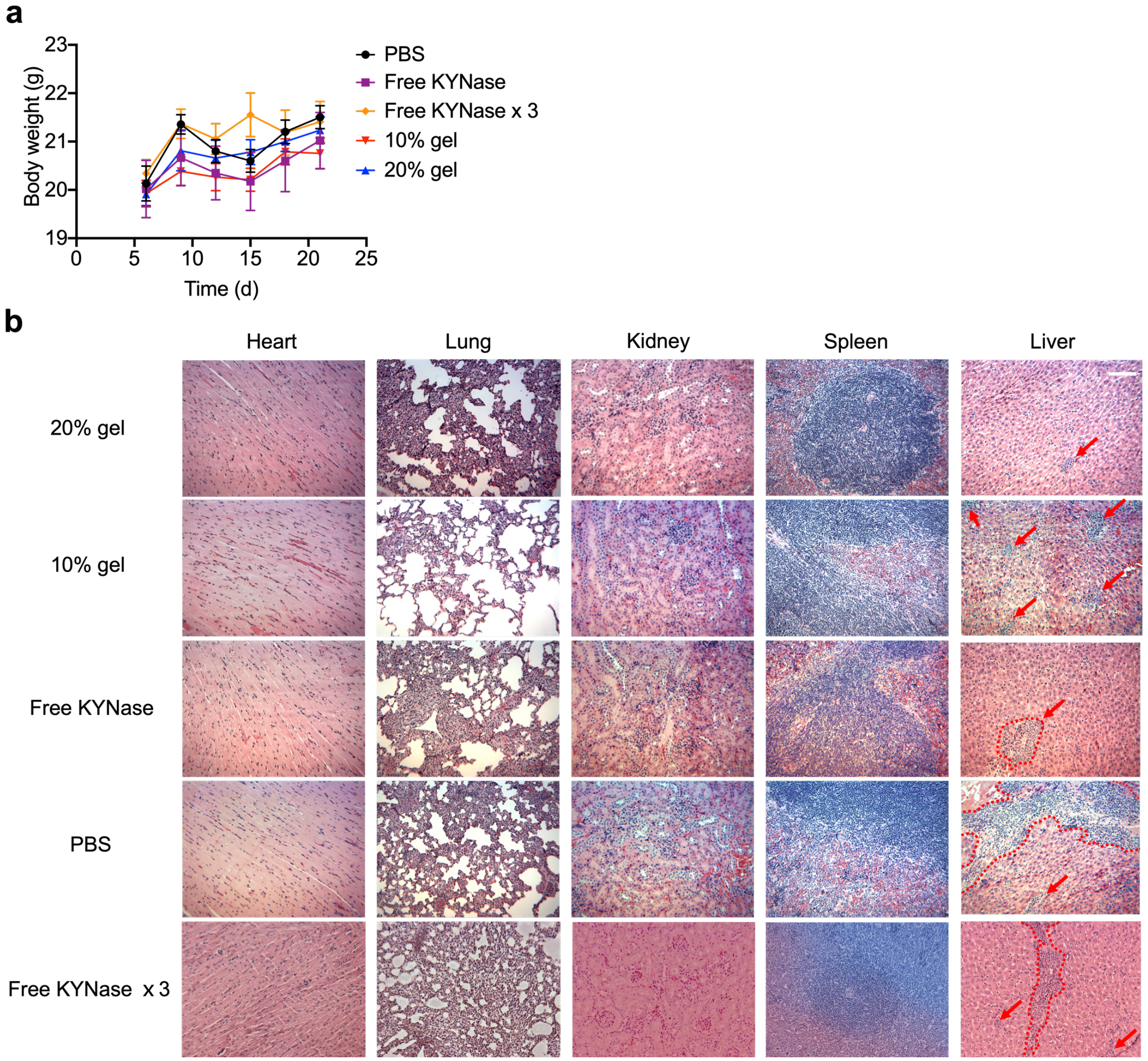

Supplementary Fig. 7. The hydrogel loaded with KYNase, but not deactivated KYNase, inhibits 4T1 tumor growth. 4T1 tumor cells were injected subcutaneously on day 0. Mice were treated with PBS, KYNase-loaded 20% hydrogel, and heat-deactivated KYNase-loaded 20% hydrogel, respectively, on day 6 when tumor volumes reached 50mm^3. Average tumor growth over time is presented. n = 5 mice for PBS, n = 6 mice for hydrogel groups. Data are represented as mean ± s.e.m., and statistical significance was determined by two-way ANOVA with Turkey's post hoc test. Source data are provided as a Source Data file.

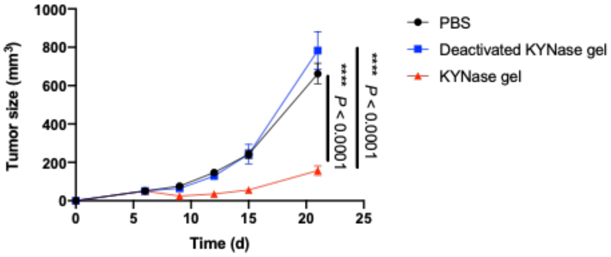

Supplementary Fig. 8. Dox release profile from 20% hydrogel in 4T1 tumor-bearing mice. n = 4 biologically independent samples. Data are presented as mean ± s.d.. Source data are provided as a Source Data file.

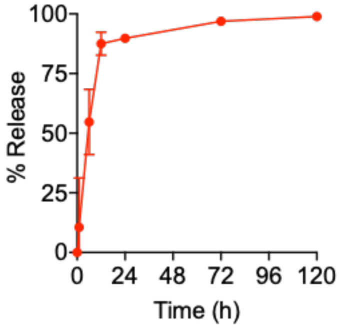

Supplementary Fig. 9. Histological analysis of tumor tissues on day 25 from mice that received different treatments, with enlargements of the regions indicated by dashed lines, related to Fig. 3. n = 5 mice for PBS and n = 7 mice for the other groups. Representative images from one mouse were shown. Scale bar, 100μm. The experiment was performed once.

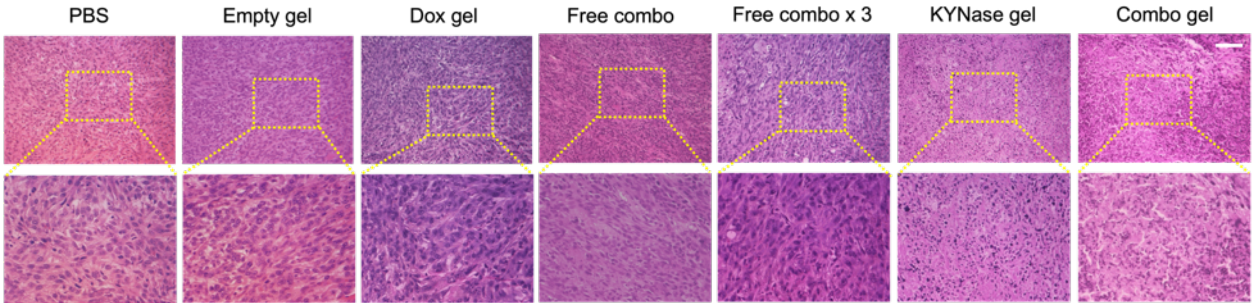

Supplementary Fig. 10. Safety of KYNase- and Dox-loaded combination hydrogel for 4T1 tumor treatment, related to Fig. 3. a, Body weight changes of different treatment groups of mice. **b,** H&E staining of major organs from differently treated mice harvested on day 25. n = 5 mice for PBS and n = 7 mice for the other groups. Representative images from one mouse were shown. Red arrows indicate metastasis. Scale bar, 100μm. Data are mean ± s.e.m.. The experiment was performed once. Source data are provided as a Source Data file.

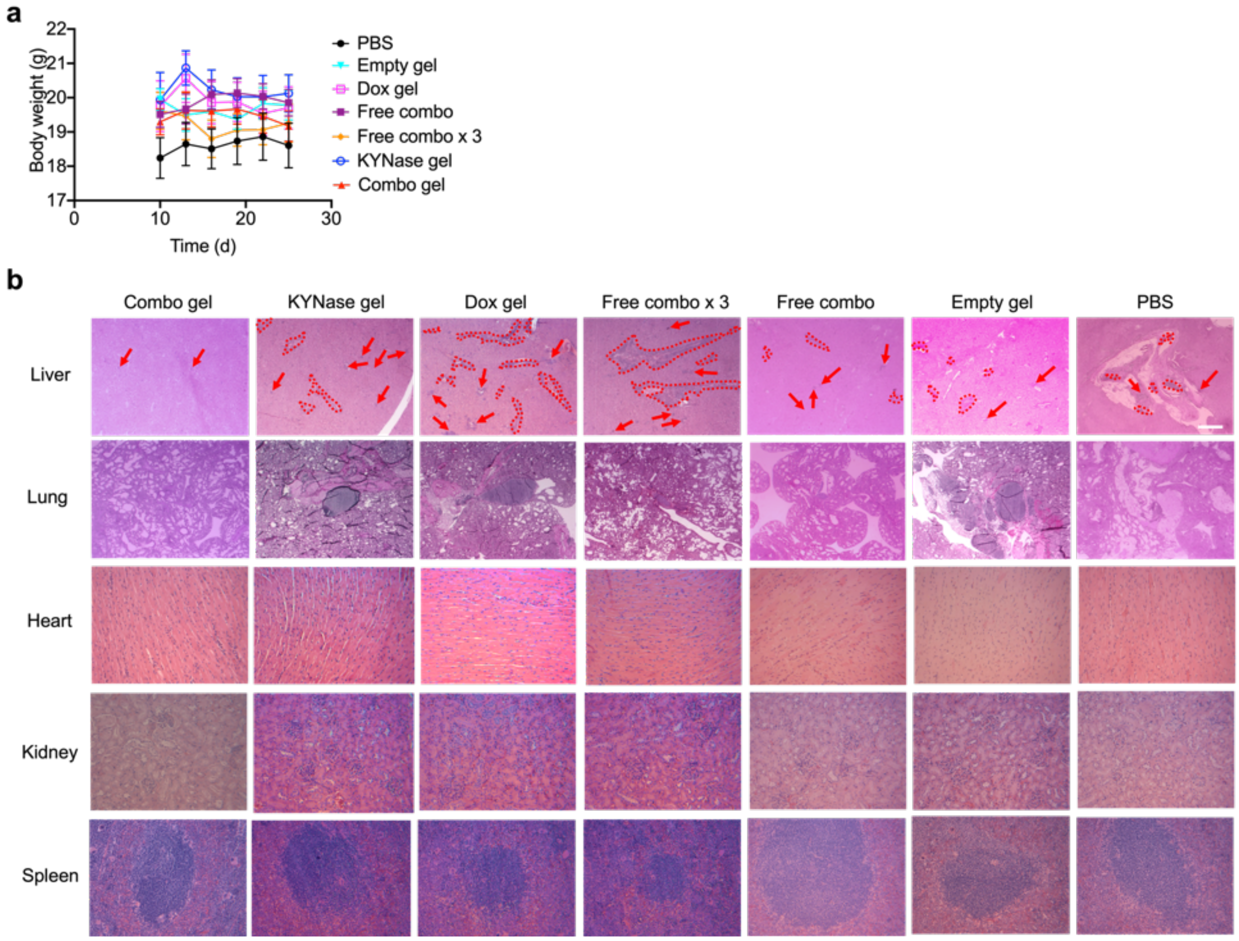

Supplementary Fig. 11. Characterization of TILs in 4T1 tumor tissues after different treatments, related to Fig. 3. a, b, Representative FACS plots of IFNγ$^+$TNFα$^+$ (a) and IL2$^+$ (b) CD8$^+$ T cells after *ex vivo* stimulation. **c, d,** Representative FACS plots (left) and quantification (right) of IFNγ$^+$TNFα$^+$ (c) and IL2$^+$ (d) CD4$^+$ T cells after *ex vivo* stimulation. **e,** Representative FACS plots of CD39$^+$CD8$^+$ T cells in tumor tissues. **f,** Representative FACS plots (left) and percentage (right) of T$_{reg}$s. n = 3 mice for PBS and empty hydrogel groups, n = 5 mice for the combination hydrogel group, and n = 4 mice for the other groups. Data are represented as mean ± s.e.m., and statistical significance was determined by one-way ANOVA with Turkey's post hoc test. Source data are provided as a Source Data file.

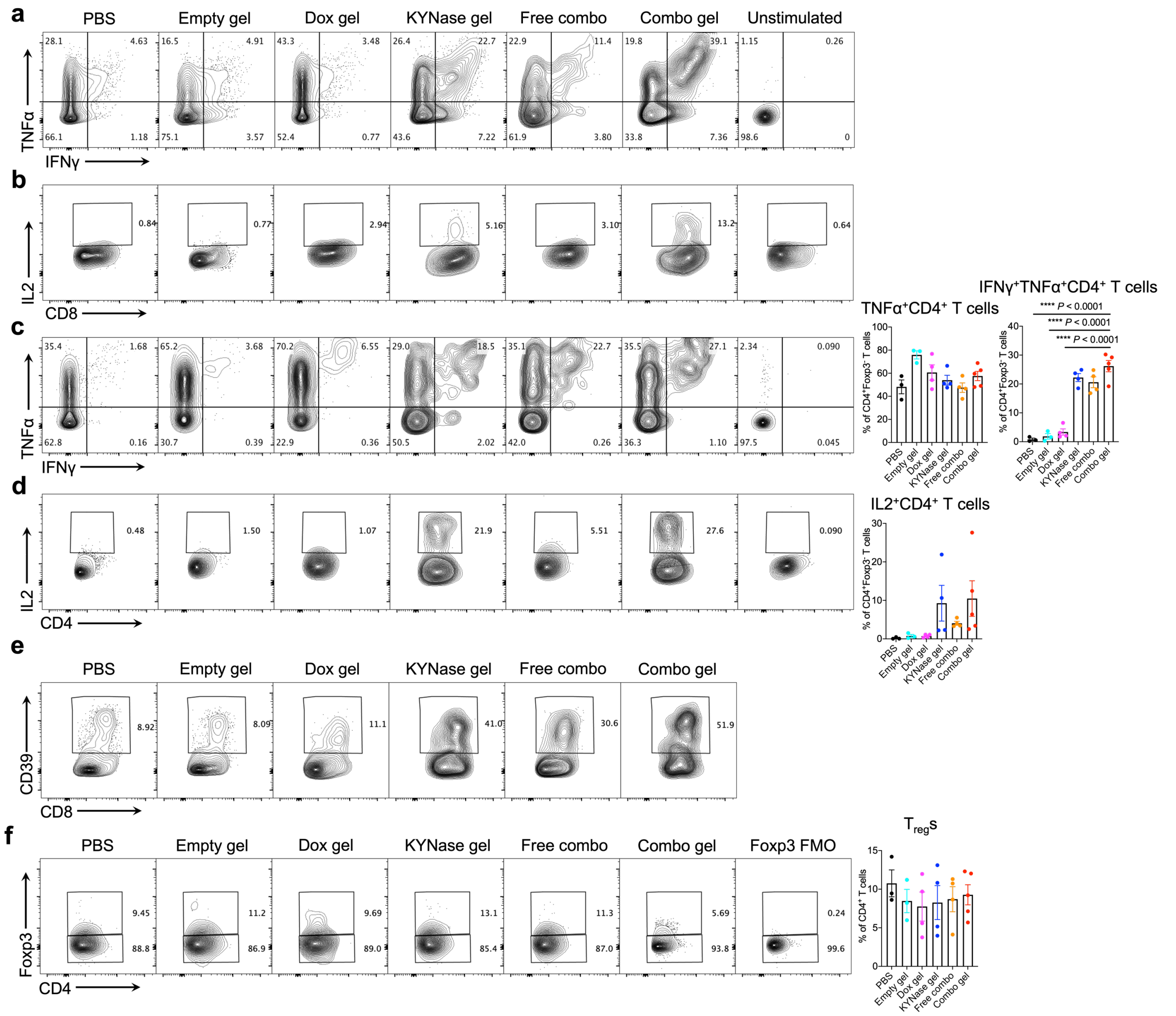

Supplementary Fig. 12. Characterization of 4T1 tumor-infiltrating myeloid lineages after different treatments, related to Fig. 3. a, b, Representative FACS plots of CD86$^+$ DCs (a) and M1-like macrophages (b). **c, d,** Representative FACS plots (left) and quantification (right) of M2-like macrophages (c) pre-gated on CD45$^+$CD11b$^+$F4/80$^+$ populations, and MDSCs (d) in tumor tissues. n = 3 mice for PBS and empty hydrogel groups, n = 5 mice for the combination hydrogel group, and n = 4 mice for the other groups. Data are represented as mean ± s.e.m.. Source data are provided as a Source Data file.

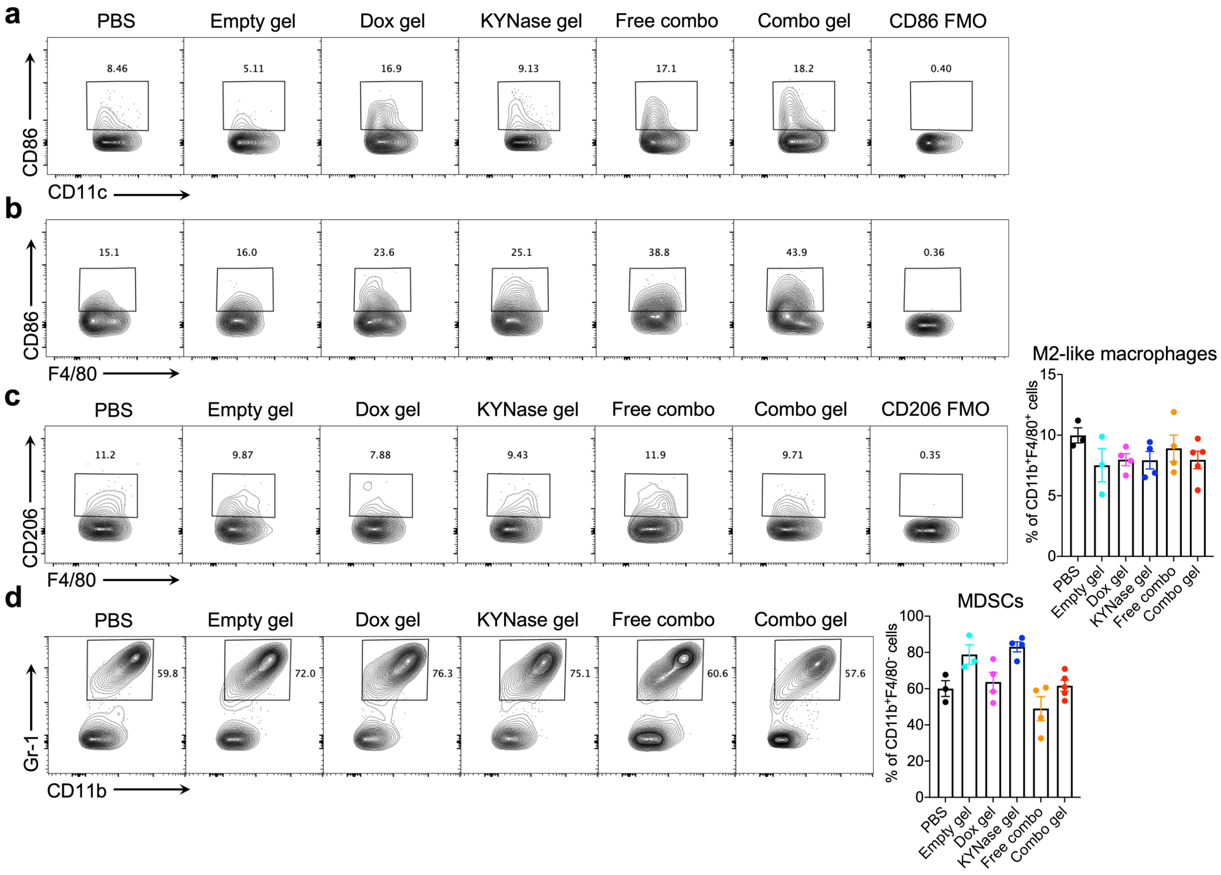

Supplementary Fig. 13. Characterization of 4T1 tumor cells after different treatments, related to Fig. 3. Representative FACS plots of PD-L1⁺ (a) and calreticulin⁺ (b) tumor cells.

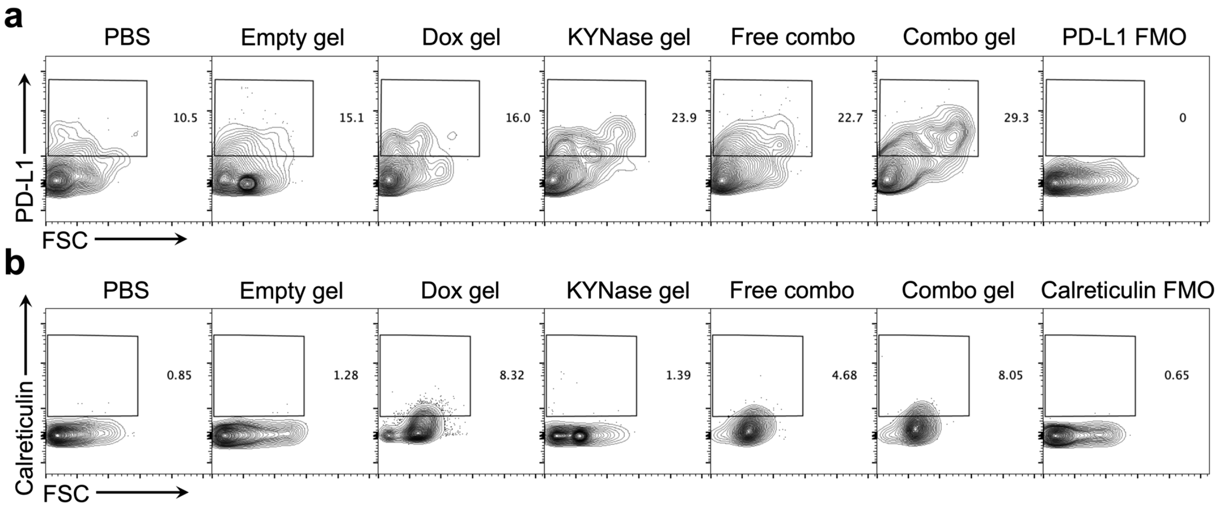

Supplementary Fig. 14. Characterization of TdLNs after different treatments, related to Fig. 4. a, Representative FACS plots (left) and quantification (right) of IFNγ$^+$CD4$^+$ T cells after restimulation with 4T1 tumor cells. **b,** Representative FACS plots of CD86$^+$ DCs in TdLNs. n = 3 mice for PBS and empty hydrogel groups, n = 5 mice for the combination hydrogel group, and n = 4 mice for the other groups. Data are represented as mean ± s.e.m., and statistical significance was determined by one-way ANOVA with Turkey's post hoc test. Source data are provided as a Source Data file.

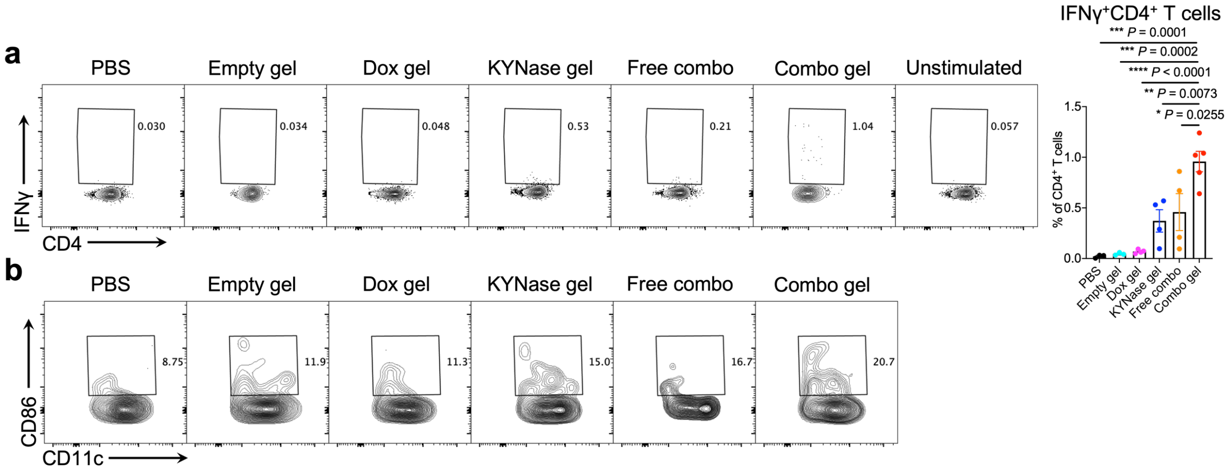

Supplementary Fig. 15. Characterization of M2-like macrophages in tumor tissues and T_{EM}s in spleens, related to Fig. 5. a, Percentage of M2-like macrophages pre-gated on $CD45^+CD11b^+F4/80^+$ populations. **b, c,** Representative FACS plots (left) and quantification (right) of $CD8^+$ (b) and $CD4^+$ (c) T_{EM}s ($CD44^{high}CD62L^{low}$) in the spleens of mice on day 25. n = 3 mice for PBS and n = 4 mice for the other groups. Data are presented as mean ± s.e.m., and one-way ANOVA with Turkey's post hoc test was performed. Source data are provided as a Source Data file.

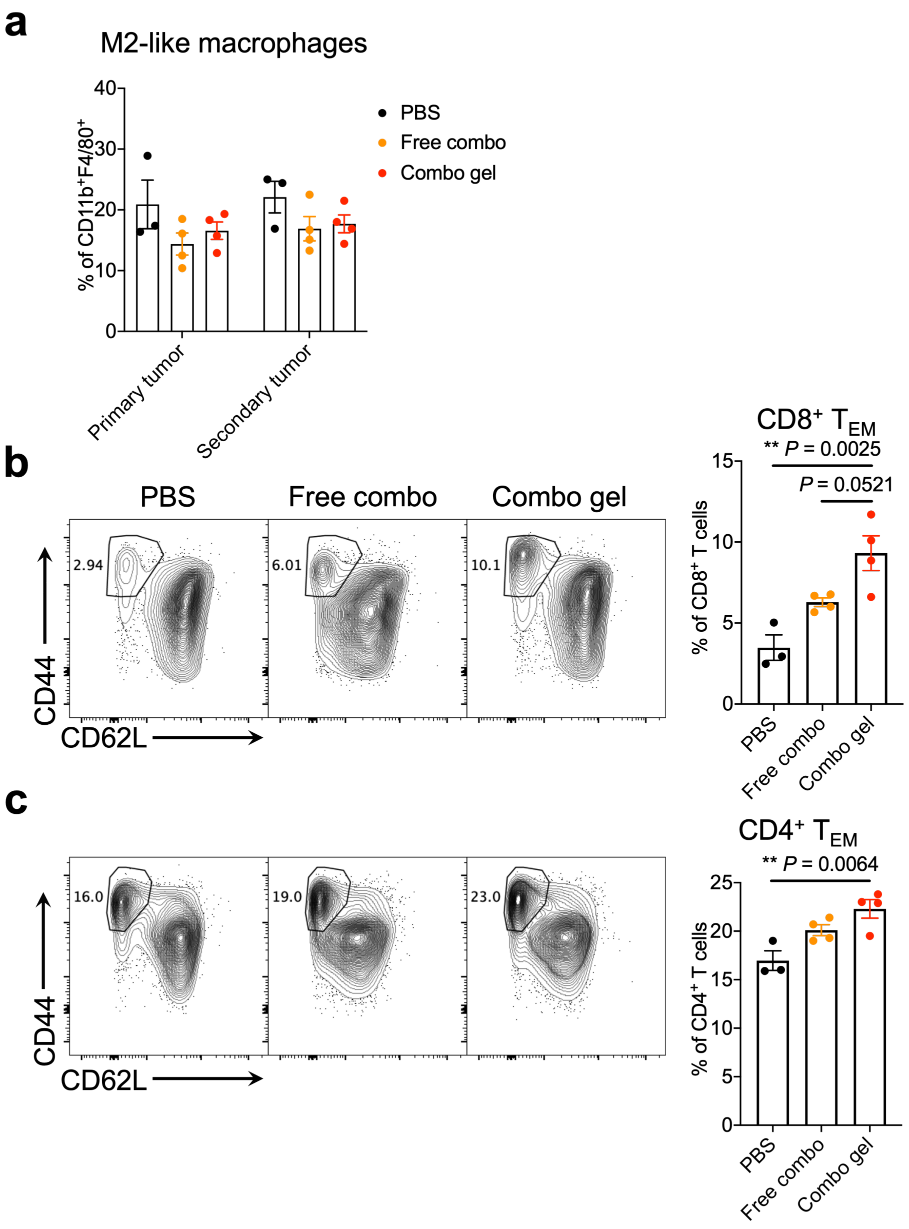

Supplementary Fig. 16. Body weight changes of B16F10 tumor-bearing mice receiving different treatments, related to Fig. 6. n = 5 mice for PBS and free combination, and n = 6 mice for combination hydrogel. Data are presented as mean ± s.e.m.. Source data are provided as a Source Data file.

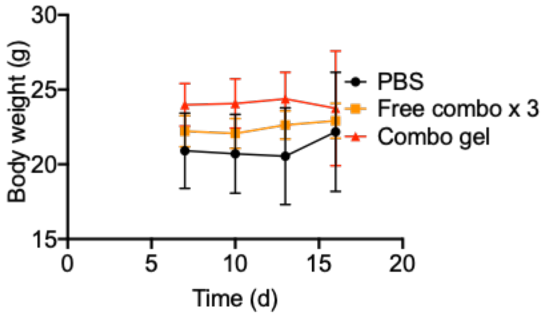

Supplementary Fig. 17. Characterization of T_{reg}s, M2-like macrophages, and MDSCs in tumor tissues and B16F10-specific $CD4^+$ T cells in TdLNs, related to Fig. 6. a-c, Quantification of T_{reg}s (a), M2-like macrophages pre-gated on $CD45^+CD11b^+F4/80^+$ cells (b), and MDSCs (c) in tumor tissues. **d,** Percentage of $IFN\gamma^+CD4^+$ T cells in TdLNs after restimulation with B16F10 tumor cells. n = 3 mice for PBS, n = 4 mice for free combination group, and n = 5 mice for combination hydrogel group. Data are presented as mean ± s.e.m.. Source data are provided as a Source Data file.

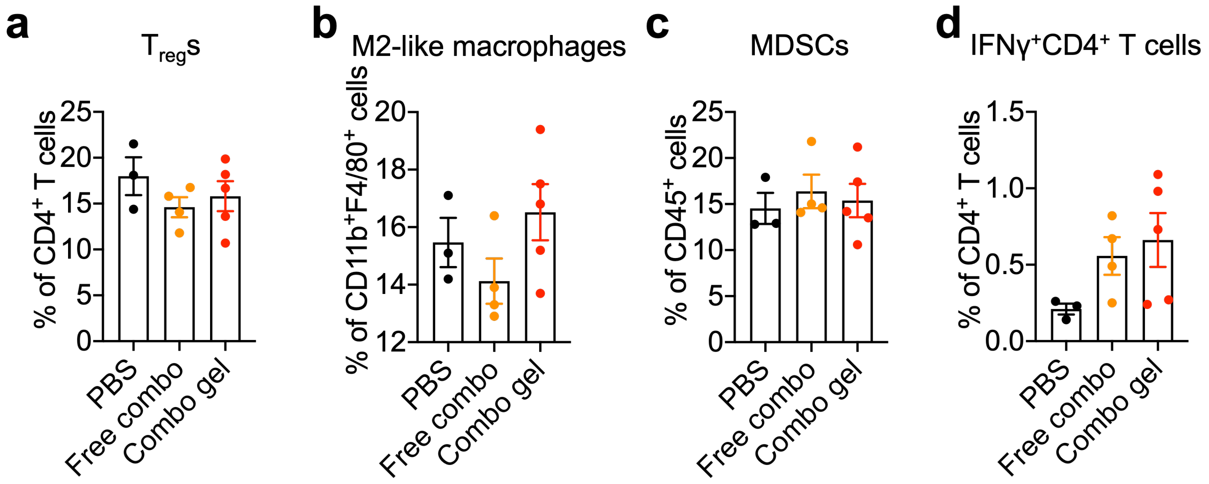

Supplementary Fig. 18. Gating strategies for FACS analysis of tumor-infiltrating leukocytes. a, Analysis of TILs. Quantitative analysis in Fig. 3e, f, g, and h use gating vi, vii and ix, xii, and viii, respectively. Quantitative analysis in Supplementary Fig. 11c, d, and f use gating xii, xi, and x, respectively. **b,** Analysis of myeloid lineages. Quantitative analysis in Fig. 3i and j use gating vi and ix, respectively. Quantitative analysis in Supplementary Fig. 12c and d use gating x and viii, respectively.

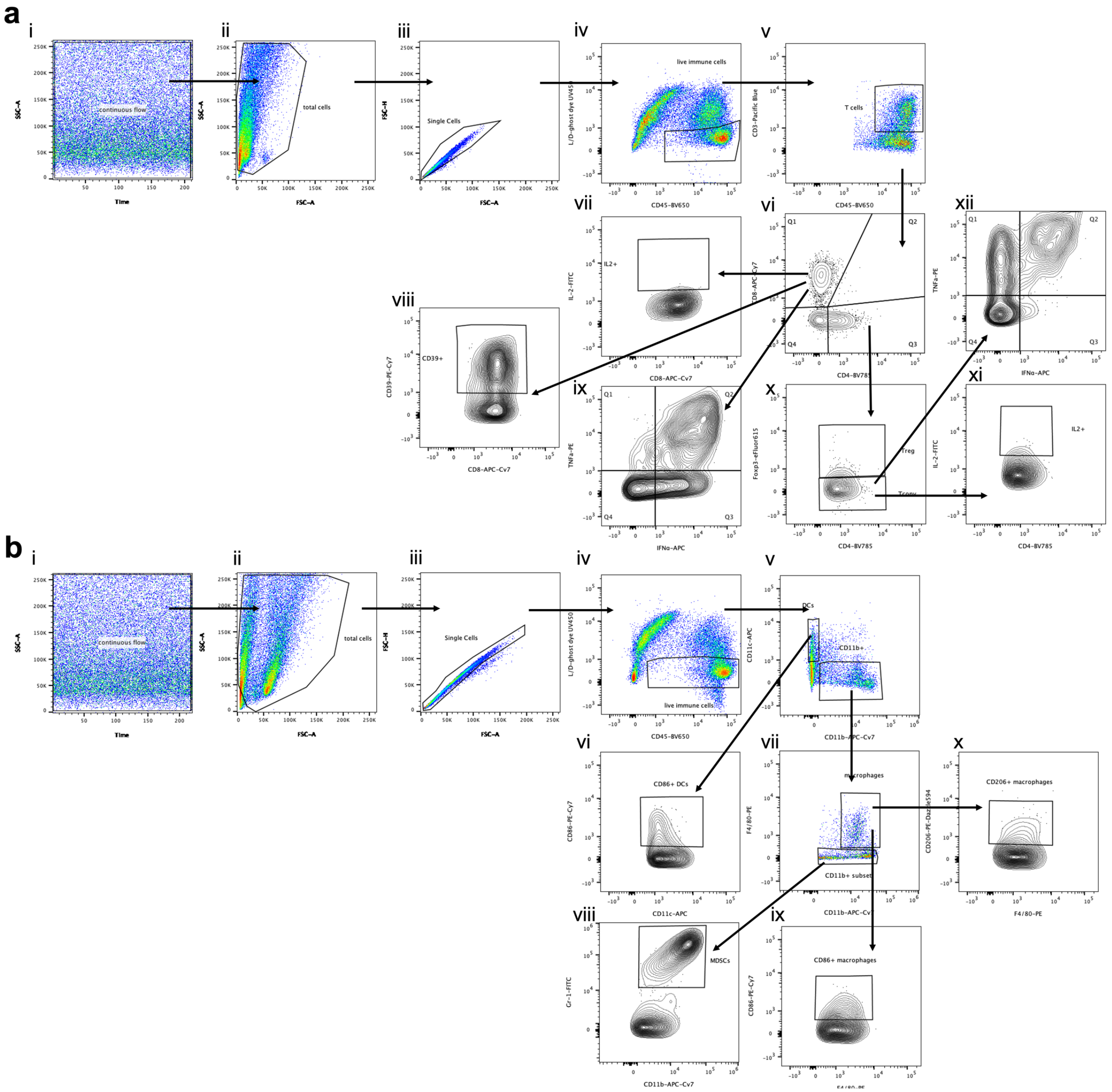

Supplementary Fig. 19. Gating strategies for FACS analysis of tumor cells. Quantitative analysis in Fig. 3k use gating v and vi.

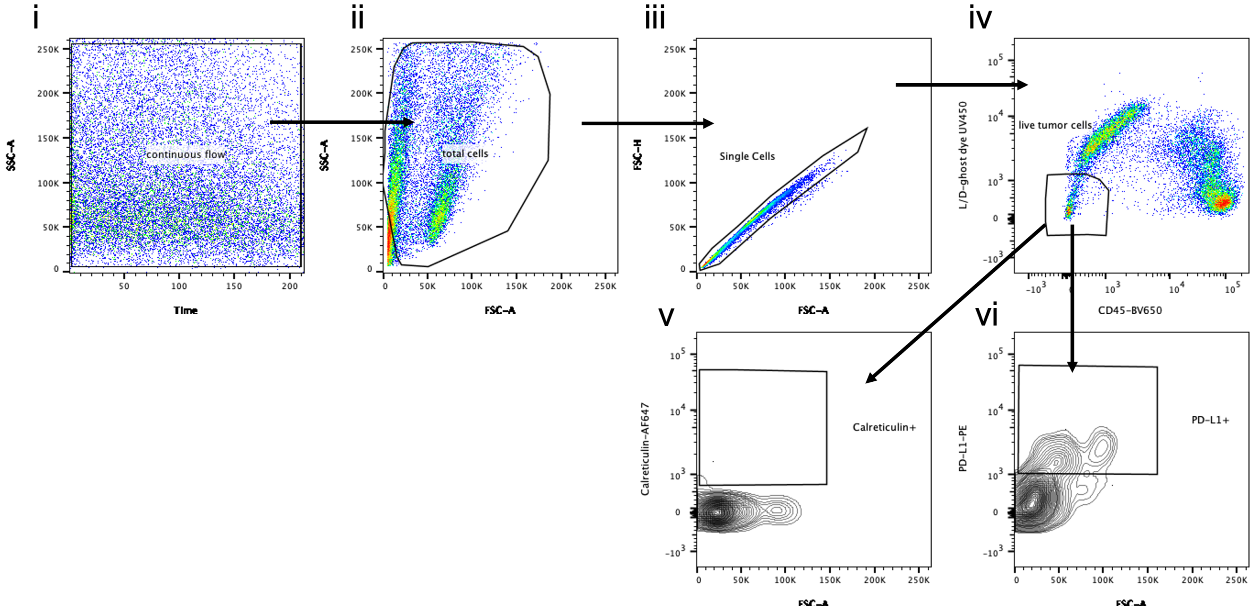

Supplementary Fig. 20. Gating strategies for FACS analysis of TdLNs. Quantitative analysis in Fig. 4b and c use gating viii and x, respectively. Quantitative analysis in Supplementary Fig. 14a use gating ix.

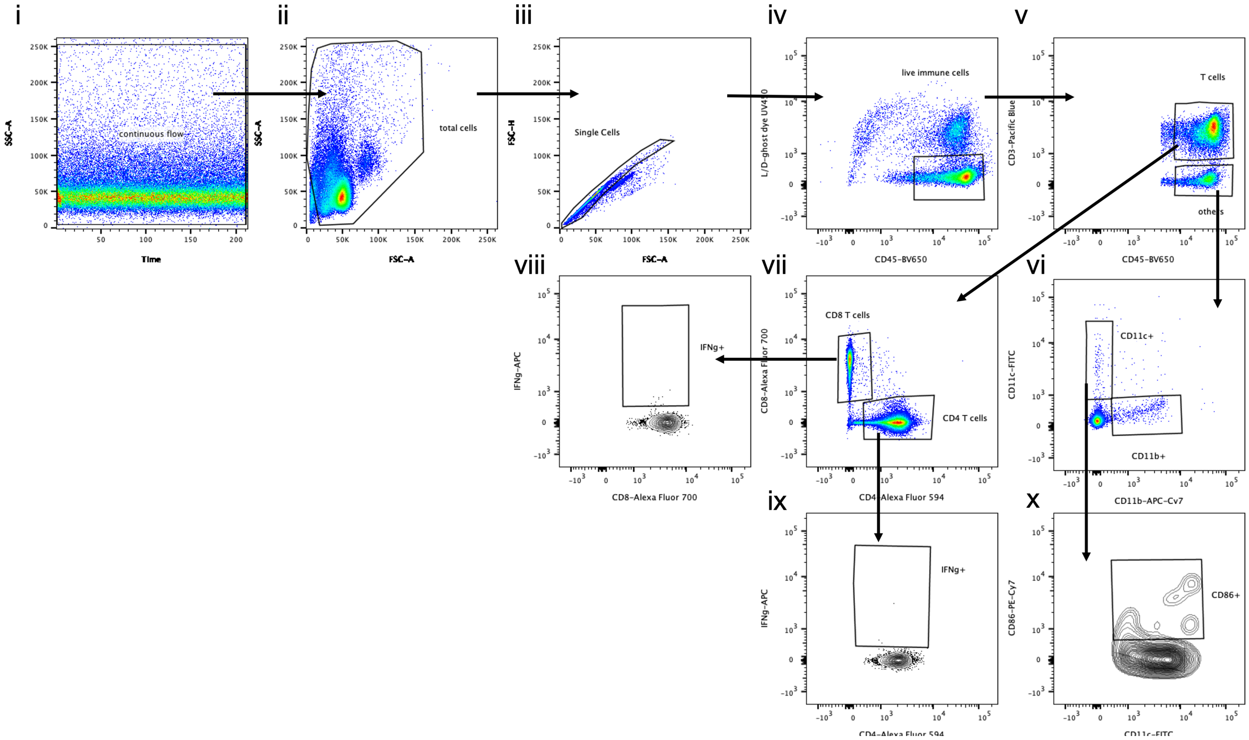

Supplementary Fig. 21. Gating strategies for FACS analysis of spleens. Quantitative analysis in Supplementary Fig. 15b and c use gating vii and viii, respectively.

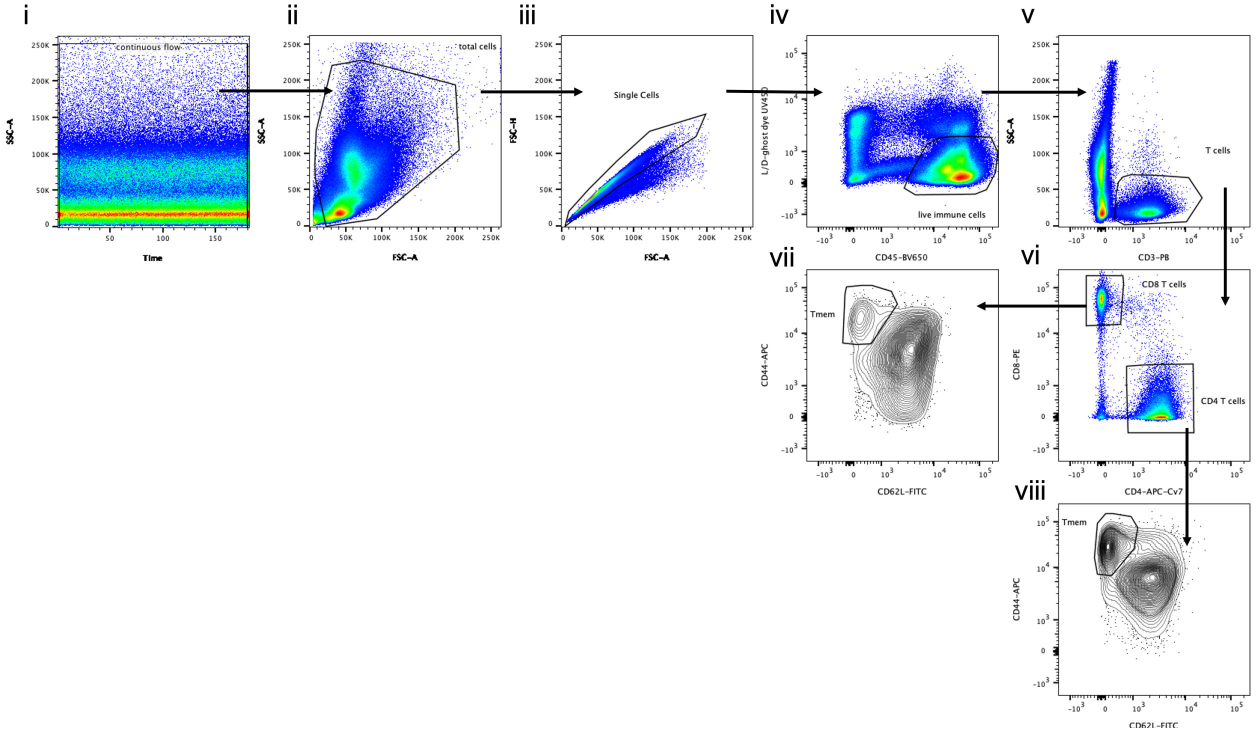

Supplement: Supplementary file 1 — Supplementary Information [file 41467_2022_31579_MOESM1_ESM.pdf]
