# Supplementary material for: An in situ hydrogel-mediated chemo-immunometabolic cancer therapy
Source: Nat Commun. 2022 Jul 2;13:3821. doi: 10.1038/s41467-022-31579-8 (PMC9250515; doi:10.1038/s41467-022-31579-8)
Supplement: Supplementary file 3 — Reporting Summary [file 41467_2022_31579_MOESM3_ESM.pdf]

nature portfolio

Corresponding author(s): Minglin Ma, Bo Wang

Last updated by author(s): Jun 13, 2022

Reporting Summary

Nature Portfolio wishes to improve the reproducibility of the work that we publish. This form provides structure for consistency and transparency in reporting. For further information on Nature Portfolio policies, see our Editorial Policies and the Editorial Policy Checklist.

Statistics

For all statistical analyses, confirm that the following items are present in the figure legend, table legend, main text, or Methods section.

n/a	Confirmed	
☐	☒	The exact sample size (*n*) for each experimental group/condition, given as a discrete number and unit of measurement
☐	☒	A statement on whether measurements were taken from distinct samples or whether the same sample was measured repeatedly
☐	☒	The statistical test(s) used AND whether they are one- or two-sided<br>*Only common tests should be described solely by name; describe more complex techniques in the Methods section.*
☐	☒	A description of all covariates tested
☐	☒	A description of any assumptions or corrections, such as tests of normality and adjustment for multiple comparisons
☐	☒	A full description of the statistical parameters including central tendency (e.g. means) or other basic estimates (e.g. regression coefficient) AND variation (e.g. standard deviation) or associated estimates of uncertainty (e.g. confidence intervals)
☒	☐	For null hypothesis testing, the test statistic (e.g. *F*, *t*, *r*) with confidence intervals, effect sizes, degrees of freedom and *P* value noted<br>*Give P values as exact values whenever suitable.*
☒	☐	For Bayesian analysis, information on the choice of priors and Markov chain Monte Carlo settings
☒	☐	For hierarchical and complex designs, identification of the appropriate level for tests and full reporting of outcomes
☒	☐	Estimates of effect sizes (e.g. Cohen's *d*, Pearson's *r*), indicating how they were calculated

Our web collection on statistics for biologists contains articles on many of the points above.

Software and code

Policy information about availability of computer code

Data collection	BD FACSDiva 8.0.1 was used for flow cytometric data collection.
Data analysis	Flowjo 10.7.1 was used for flow cytometric analysis. GraphPad Prism 9 was used for statistical analysis. GEPIA2 was used for correlation analysis between IDO1 and TDO2 expressions and CD3E and CD8A expressions in human tumors.

For manuscripts utilizing custom algorithms or software that are central to the research but not yet described in published literature, software must be made available to editors and reviewers. We strongly encourage code deposition in a community repository (e.g. GitHub). See the Nature Portfolio guidelines for submitting code & software for further information.

Data

Policy information about availability of data

All manuscripts must include a data availability statement. This statement should provide the following information, where applicable:

- Accession codes, unique identifiers, or web links for publicly available datasets
- A description of any restrictions on data availability
- For clinical datasets or third party data, please ensure that the statement adheres to our policy

All data generated from this study are available within the paper and its Supplementary Information. Human tumor data were obtained from TCGA databases. Source data are provided with this paper.

Field-specific reporting

Please select the one below that is the best fit for your research. If you are not sure, read the appropriate sections before making your selection.

- [x] Life sciences
- [ ] Behavioural & social sciences
- [ ] Ecological, evolutionary & environmental sciences

For a reference copy of the document with all sections, see nature.com/documents/nr-reporting-summary-flat.pdf

Life sciences study design

All studies must disclose on these points even when the disclosure is negative.

Sample size	Samples sizes for in vivo experiments were chosen empirically based upon preliminary tumor therapy experiments and prior knowledge of tumor challenges. Input and approval from Cornell University's Institutional Animal Care and Use Committee were also considered. In general, 3-7 biologically independent animals per group were used.<br>Sample sizes for in vitro experiments were also chosen empirically based upon preliminary experiments to achieve statistical significance. In general, 3-4 biologically independent samples per group were used.
Data exclusions	None.
Replication	In vivo experiments were repeated at least twice. SDS-PAGE and histology were performed once. Kinetic analysis were repeated once. Rheology experiments were repeated once. All replications were successful and consistent.
Randomization	Mice were age, gender, and genetic background matched. They were randomized for matched tumor sizes before treatment. In vitro experiments were not randomized as samples were tested in well-controlled conditions.
Blinding	No blinding was performed due to requirements for treatment group labeling and the predetermined nature of measurements.

Reporting for specific materials, systems and methods

We require information from authors about some types of materials, experimental systems and methods used in many studies. Here, indicate whether each material, system or method listed is relevant to your study. If you are not sure if a list item applies to your research, read the appropriate section before selecting a response.

Materials & experimental systems

n/a	Involved in the study	
[ ]	[x]	Antibodies
[ ]	[x]	Eukaryotic cell lines
[x]	[ ]	Palaeontology and archaeology
[ ]	[x]	Animals and other organisms
[x]	[ ]	Human research participants
[x]	[ ]	Clinical data
[x]	[ ]	Dual use research of concern

Methods

n/a	Involved in the study	
[x]	[ ]	ChIP-seq
[ ]	[x]	Flow cytometry
[x]	[ ]	MRI-based neuroimaging

Antibodies

Antibodies used	CD45-BV650 (BioLegend 103151, 30-F11, 1:200), CD3-Pacific Blue (BioLegend 100213, 17A2, 1:200), PD-L1-PE (BioLegend 124307, 10F.9G2, 1:100), Calreticulin-Alexa Fluor 647(R&D Systems IC38981R-100UG, 1:100), CD62L-FITC (BioLegend 104405, MEL-14, 1:200), CD8-PE (BioLegend 100707, 53-6.7, 1:200), CD8-APC-Cy7 (BioLegend 100713, 53-6.7, 1:200), CD8-Alexa Fluor 700 (BioLegend 100729, 53-6.7, 1:200), CD44-APC (BioLegend 103011, IM7, 1:200), CD4-APC-Cy7 (Tonbo Biosciences 25-0041-U025, GK1.5, 1:200), CD4-BV785 (BioLegend 100551, RM4-5, 1:200), CD4-AlexaFluor 594 (BioLegend 100446, GK1.5, 1:200), IL2-FITC (BioLegend 503805, JES6-5H4, 1:100), TNFα-PE (BioLegend 506305, MP6-XT22, 1:100), Foxp3-eFluor615 (eBioscience 42-5773-82, FJK-16s, 1:100), CD39-PE-Cy7 (BioLegend 143805, Duha59, 1:200), IFNγ-APC (BioLegend 505809, XMG1.2, 1:100), CD11c-FITC (BioLegend 117305, N418, 1:200), CD11c-APC (BioLegend 117309, N418, 1:200), F4/80-PE (BioLegend 123109, BM8, 1:200), CD86-PE-Cy7 (BioLegend 105013, GL-1, 1:200), CD11b-APC-Cy7 (BioLegend 101225, M1/70, 1:200), Gr-1-FITC (Tonbo Biosciences 35-5931-U025, RB6-8C5, 1:200), and CD206-PE-Dazzle594 (BioLegend 141731, C068C2, 1:200)
Validation	The antibody validation is provided on the manufacturers' websites.<br>CD45-BV650: https://www.biolegend.com/en-us/products/brilliant-violet-650-anti-mouse-cd45-antibody-11987<br>CD3-Pacific Blue: https://www.biolegend.com/en-us/products/pacific-blue-anti-mouse-cd3-antibody-3317<br>PD-L1-PE: https://www.biolegend.com/en-us/products/pe-anti-mouse-cd274-b7-h1-pd-l1-antibody-4497<br>Calreticulin-Alexa Fluor 647: https://www.rndsystems.com/products/human-calreticulin-alexa-fluor-647-conjugated-antibody-681233_ic38981r<br>CD62L-FITC: https://www.biolegend.com/en-us/products/fitc-anti-mouse-cd62l-antibody-384<br>CD8-PE: https://www.biolegend.com/en-us/products/pe-anti-mouse-cd8a-antibody-155

CD8-APC-Cy7: https://www.biolegend.com/en-us/products/apc-cyanine7-anti-mouse-cd8a-antibody-2269
CD8-Alexa Fluor 700: https://www.biolegend.com/en-us/products/alexa-fluor-700-anti-mouse-cd8a-antibody-3387
CD44-APC: https://www.biolegend.com/en-us/products/apc-anti-mouse-human-cd44-antibody-312
CD4-APC-Cy7: https://tonbobio.com/products/apc-cyanine7-anti-mouse-cd4-gk1-5
CD4-BV785: https://www.biolegend.com/en-us/products/brilliant-violet-785-anti-mouse-cd4-antibody-7954
CD4-AlexaFluor 594: https://www.biolegend.com/en-us/products/alexa-fluor-594-anti-mouse-cd4-antibody-9412
IL2-FITC: https://www.biolegend.com/en-us/products/pe-cyanine7-anti-mouse-il-2-antibody-8324?GroupID=GROUP24
TNFα-PE: https://www.biolegend.com/en-us/products/pe-anti-mouse-tnf-alpha-antibody-978
Foxp3-eFluor615: https://www.thermofisher.com/antibody/product/FOXP3-Antibody-clone-FJK-16s-Monoclonal/42-5773-82
CD39-PE-Cy7: https://www.biolegend.com/en-us/products/pe-cyanine7-anti-mouse-cd39-antibody-9645
IFNγ-APC: https://www.biolegend.com/en-us/products/apc-anti-mouse-ifn-gamma-antibody-993
CD11c-FITC: https://www.biolegend.com/en-us/products/fitc-anti-mouse-cd11c-antibody-1815
CD11c-APC: https://www.biolegend.com/en-us/products/apc-anti-mouse-cd11c-antibody-1813
F4/80-PE: https://www.biolegend.com/en-us/products/pe-anti-mouse-f4-80-antibody-4068
CD86-PE-Cy7: https://www.biolegend.com/en-us/products/pe-cyanine7-anti-mouse-cd86-antibody-3046
CD11b-APC-Cy7: https://www.biolegend.com/en-us/products/apc-cyanine7-anti-mouse-human-cd11b-antibody-3930
Gr-1-FITC: https://tonbobio.com/products/fitc-anti-mouse-ly-6g-gr-1-rb6-8c5
CD206-PE-Dazzle594: https://www.biolegend.com/en-us/products/pe-dazzle-594-anti-mouse-cd206-mmr-antibody-12093

Eukaryotic cell lines

Policy information about cell lines

Cell line source(s)	4T1 breast cancer and B16F10 melanoma cell lines were obtained from X. Wang and C. Kenific (Lyden lab) at Cornell University. They were originally obtained from ATCC (CRL-2539 and CRL-6475).
Authentication	4T1 and B16F10 cell lines were previously reported and commonly used. They were not authenticated by us.
Mycoplasma contamination	All cell lines were negative for mycoplasma.
Commonly misidentified lines (See ICLAC register)	None.

Animals and other organisms

Policy information about studies involving animals; ARRIVE guidelines recommended for reporting animal research

Laboratory animals	Male and female C57BL/6 and female BALB/c mice were purchased from the Jackson Laboratory and were 6-10 weeks old at the beginning of each experiment. The mice were housed on a 12h light-dark cycle at 20-26°C with 30-70% humidity.
Wild animals	None.
Field-collected samples	None.
Ethics oversight	All animal studies were performed following the protocols approved by Cornell University's Institutional Animal Care and Use Committee and in compliance with all relevant ethical regulations.

Note that full information on the approval of the study protocol must also be provided in the manuscript.

Flow Cytometry

Plots

Confirm that:

- [x] The axis labels state the marker and fluorochrome used (e.g. CD4-FITC).
- [x] The axis scales are clearly visible. Include numbers along axes only for bottom left plot of group (a 'group' is an analysis of identical markers).
- [x] All plots are contour plots with outliers or pseudocolor plots.
- [x] A numerical value for number of cells or percentage (with statistics) is provided.

Methodology

Sample preparation	Tumors were cut into small pieces and digested in dissociation buffer (RPMI 1640, 1mg/ml collagenase IV (Worthington Biochemical Corporation), and 100µg/ml DNAse I (Thermo Scientific)) at 37°C for 45min with gentle shaking. The cell suspension was passed through a 70µm cell strainer and treated with ACK lysing buffer (Gibco). TdLNs were mechanically digested and passed through a 70µm cell strainer to obtain single cell suspensions. Spleens were mechanically disrupted, filtered through a 70µm cell strainer, and treated with ACK lysing buffer to obtain single cell suspensions.
Instrument	A BD FACSymphony A3 was used for flow cytometric data acquisition.

March 2021

Software	BD FACSDiva 8.0.1 was used for data collection, and data were analyzed with Flowjo 10.7.1.
Cell population abundance	The abundance of populations of interest was determined by appropriate gating and reported.
Gating strategy	After appropriate compensation was made using single color compensation controls, gates were set using naive mice spleen control, FMO (full-minus one) stains, or isotype control antibodies.

☒ Tick this box to confirm that a figure exemplifying the gating strategy is provided in the Supplementary Information.
